# Phase Behaviors of ABA Star Polymer and Nanoparticles Confined in a Sphere with Soft Inner Surface

**DOI:** 10.3390/polym14081610

**Published:** 2022-04-15

**Authors:** Minna Sun, Zhiwei Zhang, Ying Li, Wen Li, Qingwei Liao, Lei Qin

**Affiliations:** 1 Beijing Key Laboratory for Sensors, Beijing Information Science and Technology University, Beijing 100192, China; sunminna331@163.com (M.S.); zhiweizhang@bistu.edu.cn (Z.Z.); liying@bistu.edu.cn (Y.L.); liaoqingwei@bistu.edu.cn (Q.L.); 2 Beijing Key Laboratory for Optoelectronic Measurement Technology, Beijing Information Science and Technology University, Beijing 100192, China; 17864730350@163.com; 3 Department of Pharmacy, Changzhi Medical College, Changzhi 046000, China

**Keywords:** phase behaviors, self-consistent field theory, nanoparticles, grafted polymers, star polymer

## Abstract

The phase behaviors of an ABA star polymer and nanoparticles confined in a sphere with soft inner surface, which is grafted with homopolymer brushes have been studied by the self-consistent field theory (SCFT). The morphologies of mixture in the center slice of sphere were focused. Two cases are considered: one is that the nanoparticles interact with the B blocks and the other is that the nanoparticles preferentially wet the B blocks. Under the two conditions, through changing the block ratio of the ABA star polymer, the concentration and radius of the nanoparticles, the phase behaviors of the mixtures confined the soft sphere are studied systematically. With increasing the concentration of nanoparticles, the entropy and the steric repulsive interaction of nanoparticles, and the nanoparticle density distributions along the perpendicular line through the center of sphere are plotted. The phase diagram is also constructed to analyze the effects of the nanoparticle volume fraction and radius on morphologies of ABA star polymers, and to study the effect of confinement on the phase behaviors. The results in this work provide a useful reference for controlling the ordered structures in experiment, which is an effective way to fabricate the newly multifunctional materials.

## 1. Introduction

The mixture of organic copolymers and inorganic nanoparticles can form a series of ordered structures either in bulk or under confinements, which have attracted considerable attentions in recent years. These novel structures have a wide range of applications [1,2,3,4,5,6], such as biological [7,8,9,10,11,12,13], electronic [14,15], photonic [16,17,18], mechanical [19,20,21,22,23] and catalytic materials [24,25]. Huh et al. had studied the morphologies of diblock copolymer-particle mixtures with the nanoparticles in theory [26], which can form novel structures. Lee et al. had investigated the influence of nanoparticles on the morphologies of diblock copolymer and permitted it to interact with both the A and B blocks [27]. By comparing the two conditions, they found the variations of the interactions between nanoparticles and blocks were able to affect the phase transitions of the mixtures. Also, the sizes and chemistry of the particles could significantly affect the morphologies of organic-inorganic hybrid materials. For one-dimensional confinement, the copolymer-nanoparticle mixtures confined between two hard and parallel plates were studied. The polymers could drive the nanoparticles to the walls in such confined geometries and entropic effects could be harnessed in order to improve the fabrication of novel materials [28,29,30,31]. For two-dimensional confinement, the phase behaviors of copolymer-nanoparticle mixtures in nanopore confinement were studied by Yang et al. and the nanoparticles were allowed to interact with each block. In this case, the nanoparticles were located along the interface of A and B blocks, and the asymmetric diblock copolymer were found to form a mixed phase of concentric lamellae and cylinder rather than cylindrical structures in the bulk [32], which could fabricate new nanodevices. Pan et al. explored the phase behaviors of the mixture of diblock copolymer and nanoparticles confined between two concentric circular walls [33]. They pointed that the nanoparticles had a significant effect in this system that when the distance between two concentric circular walls was extremely small, the perpendicular lamellar structure could be formed, which tested and verified the view presented by Balazs [30,31]. The effect of nanoparticle shape on the mixture of block copolymers and nanoparticles were explored by Halevi et al. and the nanoparticle shape was found to be able to change the orientation of the morphologies of block copolymer-nanoparticle mixtures [34]. In addition, considerable progress has been made in controlling the nanoparticles distribution in the morphologies of block copolymers [35,36]. In this paper, the nanoparticles were allowed to interact with the B blocks, and also interact with both the A blocks and the homopolymer brushes.

To improve the properties of the materials, we tune the interfacial properties between copolymers and confined surface. Grafting the polymers on the confined surface is an useful way to tune the interfacial properties [37], which also can effectively avoid the frustrated structures and obtain more ordered structures, so we input the copolymer-nanoparticle mixtures into a geometry with soft surface [38,39,40,41]. For one-dimensional confinement, Zhou and Ma had investigated the patterns of star polymer and nanoparticles confined between two “soft” surfaces and obtained more new structures [42]. However, the morphologies of the mixture of diblock copolymer and nanoparticles under geometries with soft surface are much less explored.

To investigate the phase behaviors of the ABA star polymer-nanoparticles mixture in a sphere with soft inner surface, a developed theory is adopted [27,28,43,44], which is the combination of self-consistent field theory (SCFT) for the copolymers and density functional theory (DFT) for the nanoparticles [32,33]. The SCFT has been developed successfully to predict the morphologies of copolymers [45,46], and the DFT was successful in explaining the steric packing effect of nanoparticles [47,48]. The combination of SCFT and DFT provides an effective method for studying the morphologies of ABA star polymer and nanoparticles confined in a sphere.

## 2. Materials and Methods

**Figure 1:**
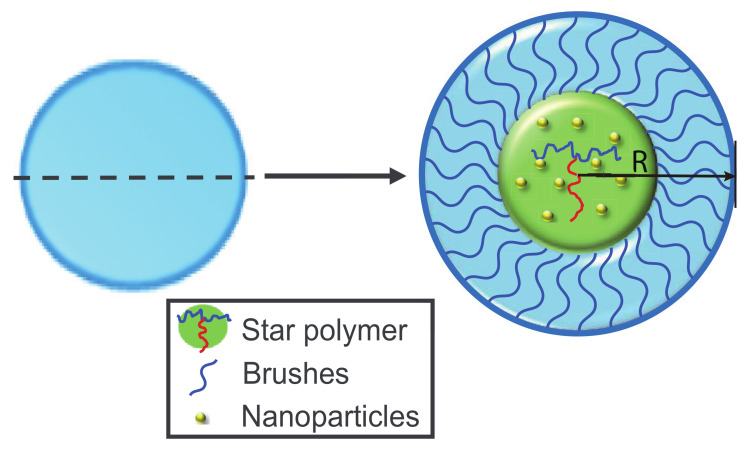
Schematic diagram of the ABA star polymer-nanoparticles mixture confined in a sphere with soft inner surface. The polymer, the nanoparticles and brushes are represented in green, gold and blue, respectively.

An incompressible mixture of $n_{p}$ nanoparticles and $n_{co}$ ABA star polymer was confined in a sphere whose inner surface was grafted by $n_{br}$ A-type homopolymer chains (Figure 1). The grafted chain ends are immobile on the substrate. The morphologies of mixture in the center slice of sphere were focused. The grafting density was defined as $\sigma =n_{br}/\left(2\pi R\right)$. Both the ABA star polymer and grafted homopolymer chains were composed of *N* segments and the statistical lengths for ABA star polymers and homopolymer were denoted with *a*. The lengths were expressed in units of the radius of gyration, $R_{g}$, of the polymers. The fractions of A and B monomer in a copolymer chain were denoted by $f_{i}(i=$ a,b) $\left(0\leq f_{i}\leq 1\right)$ and each segment had a fixed volume of 1/ρ. The Flory-Huggins interaction parameters were expressed by $\chi_{ij}(i,j=$ a, b, p, brush). $\varphi_{a}$, $\varphi_{b}$, $\varphi_{br}$ and $\varphi_{p}$ were the local volume fractions of the A blocks, B blocks, homopolymer brushes and nanoparticles, respectively. The average volume fractions of the ABA star polymer, the homopolymer brushes and nanoparticles were expressed as $\phi_{co}=n_{co}N\rho^{-1}/V$, $\phi_{br}=n_{br}N\rho^{-1}/V$ and $\phi_{p}=1-\phi_{co}-\phi_{br}$. The chemical potential fields were expressed by $\omega_{k}\left(r\right)$ (*k*=a, b, br, p) and ξ(r) is a Lagrange multiplier to ensure the incompressibility of the system. Thus, for the mixture of nanoparticles and ABA star polymer confined in a in a sphere with volume of *V* and radius of *R*, the free energy *F* can be obtained as follows:

(1) $$\begin{array}{ccc} \frac{NF}{\rho k_{B}TV} & = & -\phi_{br}\ln\left(\frac{Q_{br}}{V\phi_{br}}\right)-\phi_{co}\ln\left(\frac{Q_{co}}{V\phi_{co}}\right)\\ & & -\frac{\phi_{p}}{\alpha }\ln\left(\frac{Q_{p}\alpha }{V\phi_{p}}\right)+\frac{1}{V}\int dr[\chi_{ab}N\varphi_{a}\left(r\right)\varphi_{b}\left(r\right)\\ & & +\chi_{bbr}N\varphi_{b}\left(r\right)\varphi_{br}\left(r\right)+\chi_{ap}N\varphi_{a}\left(r\right)\varphi_{p}\left(r\right)\\ & & +\chi_{bp}N\varphi_{b}\left(r\right)\varphi_{p}\left(r\right)+\chi_{brp}N\varphi_{br}\left(r\right)\varphi_{p}\left(r\right)\\ & & -\omega_{a}\left(r\right)\varphi_{a}\left(r\right)-\omega_{b}\left(r\right)\varphi_{b}\left(r\right)-\omega_{br}\left(r\right)\varphi_{br}\left(r\right)\\ & & -\omega_{p}\left(r\right)\varphi_{p}\left(r\right)-\xi \left(r\right)(1-\varphi_{a}\left(r\right)-\varphi_{b}\left(r\right)\\ & & -\varphi_{br}\left(r\right)-\varphi_{p}\left(r\right))+\rho_{p}\Psi_{hs}\left({\bar{\varphi }}_{p}\right)], \end{array}$$

where $k_{B}$ is the Boltzmann constant and *T* is the temperature. $Q_{co}=\int drq_{k}\left(r,s\right)q_{k}^{+}\left(r,s\right)\left(k=a_{1},b_{2},b\right)$ and $Q_{br}=\int drq_{br}\left(r,s\right)q_{br}^{+}\left(r,s\right)$ are single-chain partition functions of ABA star polymer and homopolymer brushes in the mean fields. The end-segment distribution function q(r,s) is the probability of finding a polymer chain segment of contour length *s* at position r. The chain propagators q(r,s) and its conjugate $q^{+}\left(r,s\right)$ obey the following modified diffusion equations:

(2) $$\frac{\partial \;q(r,s)}{\partial \;s}=\nabla^{2}q\left(r,s\right)-\omega \left(r\right)q\left(r,s\right),$$

and

(3) $$-\frac{\partial \;q^{+}\left(r,s\right)}{\partial \;s}=\nabla^{2}q^{+}\left(r,s\right)-\omega \left(r\right)q^{+}\left(r,s\right),$$

with $\omega \left(r,s\right)=\omega_{a1}\left(r\right)$ for $0\leq s\leq f_{a1}$, $\omega \left(r,s\right)=\omega_{a2}\left(r\right)$ for $0\leq s\leq f_{a2}$ and $\omega \left(r,s\right)=\omega_{b}\left(r\right)$ for $0\leq s\leq f_{b}$. $v_{R}=\left(4/3\right)\pi R_{P}^{3}$ is the nanoparticle volume with radius $R_{p}$. $Q_{p}=\int dr\exp\left[-\omega_{p}\left(r\right)\right]$ is the partition function of a nanoparticle to the effective chemical potential field $\omega_{p}\left(r\right)$. α is the volume ratio of the nanoparticles to block copolymer chains, $\alpha =v_{R}\rho /N$. The steric free energy of the nanoparticles is $\Psi_{hs}\left({\bar{\varphi }}_{p}\right)$, which can be calculated by the Carnahan-Starling function [49], $\Psi_{hs}\left(x\right)=\left(4x-3x^{2}\right)/{\left(1-x\right)}^{2}$. The local nanoparticle volume fraction is given by

(4) $$\varphi_{p}\left(r\right)=\frac{\alpha }{v_{R}}\int_{\left|r^{{}^{\prime }}\right|<R}dr^{{}^{\prime }}\rho_{p}\left(r+r^{{}^{\prime }}\right),$$

and the weighted nanoparticle volume fraction

(5) $${\bar{\varphi }}_{p}\left(r\right)=\frac{\alpha }{v_{2R}}\int_{\left|r^{{}^{\prime }}\right|<2R}dr^{{}^{\prime }}\rho_{p}\left(r+r^{{}^{\prime }}\right),$$

where $\rho_{p}\left(r\right)$ is the nanoparticle center distribution, $v_{2R}$ is the volume of a sphere with radius $2R_{p}$.

Minimizing the free energy in Equation (1), with respect to the monomer densities and mean fields leads to the set of mean-field equations:

(6) $$\begin{array}{ccc} \varphi_{a}\left(r\right) & = & \frac{\phi_{co}V}{Q_{co}}[\int_{0}^{f_{a1}}dsq_{a1}\left(r,s\right)q_{a1}^{+}\left(r,s\right)\\ & & +\int_{0}^{f_{a2}}dsq_{a2}\left(r,s\right)q_{a2}^{+}\left(r,s\right)], \end{array}$$

(7) $$\varphi_{b}\left(r\right)=\frac{\phi_{co}V}{Q_{co}}\int_{0}^{f_{b}}dsq_{b}\left(r,s\right)q_{b}^{+}\left(r,s\right),$$

(8) $$\varphi_{br}\left(r\right)=\frac{\phi_{br}V}{Q_{br}}\int_{0}^{1}dsq_{br}\left(r,s\right)q_{br}^{+}\left(r,s\right),$$

(9) $$\rho_{p}\left(r\right)=\frac{\phi_{p}V}{\alpha Q_{p}}\exp\left[-\omega_{p}\left(r\right)\right],$$

(10) $$\omega_{a}\left(r\right)=\chi_{ab}N\varphi_{b}\left(r\right)+\chi_{ap}N\varphi_{p}\left(r\right)+\xi \left(r\right),$$

(11) $$\begin{array}{ccc} \omega_{b}\left(r\right) & = & \chi_{ab}N\varphi_{a}\left(r\right)+\chi_{bbr}N\varphi_{br}\left(r\right)\\ & & +\chi_{bp}N\varphi_{p}\left(r\right)+\xi \left(r\right), \end{array}$$

(12) $$\begin{array}{c} \omega_{br}\left(r\right)=\chi_{bbr}N\varphi_{b}\left(r\right)+\chi_{brp}N\varphi_{p}\left(r\right)+\xi \left(r\right), \end{array}$$

(13) $$\begin{array}{ccc} \omega_{p}\left(r\right) & = & \Psi_{hs}\left({\bar{\varphi }}_{p}\left(r\right)\right)+\frac{\alpha }{v_{R}}\int_{\left|r^{{}^{\prime }}\right|<R}dr^{{}^{\prime }}[\chi_{ap}N\varphi_{a}\left(r+r^{{}^{\prime }}\right)\\ & & +\chi_{bp}N\varphi_{b}\left(r+r^{{}^{\prime }}\right)+\chi_{brp}N\varphi_{br}\left(r+r^{{}^{\prime }}\right)\\ & & +\xi \left(r+r^{{}^{\prime }}\right)]+\frac{\alpha }{v_{2R}}\int_{\left|r^{{}^{\prime }}\right|<2R}dr^{{}^{\prime }}[\rho_{p}\left(r+r^{{}^{\prime }}\right)\\ & & \Psi_{hs}^{{}^{\prime }}\left({\bar{\varphi }}_{p}\left(r+r^{{}^{\prime }}\right)\right)], \end{array}$$

and

(14) $$\varphi_{a}\left(r\right)+\varphi_{b}\left(r\right)+\varphi_{br}\left(r\right)+\varphi_{p}\left(r\right)=1.$$

In this study, there were no polymers outside the sphere, so the end-segment distribution functions were set to zero. The situation of the equal interactions among A, B and brushes was mainly focused on with $\chi_{ab}N$ = $\chi_{bbr}N$ = 20.0 and the fraction of homopolymer brush chains was set to $f_{br}=0.3$. The initial condition for copolymers $q_{a1}^{+}\left(r,f_{a1}\right)=1.0,q_{a2}^{+}\left(r,f_{a2}\right)=1.0,q_{b}^{+}\left(r,f_{b}\right)=1.0$, and $q_{a1}\left(r,0\right)=q_{a2}^{+}\left(r,0\right)q_{b}^{+}\left(r,0\right)$, $q_{a2}\left(r,0\right)=q_{a1}^{+}\left(r,0\right)q_{b}^{+}\left(r,0\right)$, $q_{b}\left(r,0\right)=q_{a1}^{+}\left(r,0\right)q_{a2}^{+}\left(r,0\right)$, and the initial condition for homopolymer brushes is $q_{br}\left(r,0\right)=1\left(\left|r\right|=R\right),q_{br}\left(r,0\right)=0\left(\left|r\right|\neq R\right),q_{br}^{+}\left(r,1\right)=1$. In addition, the chain lengths of the ABA star polymer and homopolymer chains were set to N=100. For this system, the calculation was performed in a lattice of 100×100 and the chain contour length for each block was discretized into 100 segments. Periodic boundary conditions were performed on all edges of the sphere. The calculation was started using initial random values of the field. In the program, an initial value of the concentration distribution of star copolymers in the system were set due to the phase separation affected by fluctuations. It utilized the Crank-Nicholson scheme to solve diffusion equations, which had been proven under different confinement conditions [50,51]. The free energy could be obtained from Equation (1), the volume fractions were obtained from Equations (6)–(9), and the fields were calculated by Equations (10)–(13). Then updated fields were putted in Equations (2) and (3). All calculations were iterated until the free energies difference between two sequential steps was less than $10^{-10}$.

## 3. Numerical Results and Discussions

The fraction of the B blocks, the fraction and radius of the nanoparticles, the interaction between the nanoparticles and the blocks, and the radius of the sphere were systematically considered to investigate the phase behaviors of the ABA star polymer and nanoparticles confined in a sphere with soft inner surface.

### 3.1. Nanoparticles Interact with the B Blocks

In the first part, the nanoparticles were allowed to interact with the B blocks with $\chi_{bp}N=20.0,\chi_{ap}N=\chi_{brp}N=0.0$. The repulsive interaction between the nanoparticles and the B blocks promoted the compatibility of the nanoparticles with the A blocks, thus, the nanoparticles were located in the A domains. For clarity, the nanoparticles are not shown in all the morphologies of the ABA star polymer blend.

**Figure 2:**
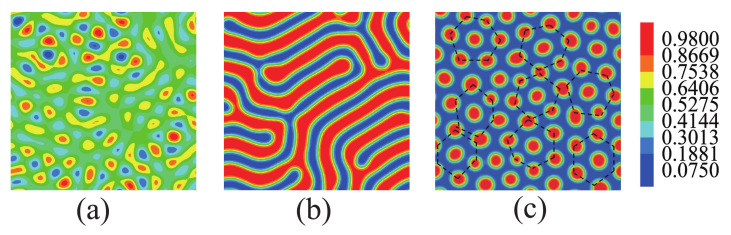
The morphologies of the ABA star polymer blend without nanoparticles. (**a**) $f_{a1}=f_{a2}=0.13,f_{b}=0.74$ (**b**) $f_{a1}=f_{a2}=0.2,f_{b}=0.6$ (**c**) $f_{a1}=f_{a2}=0.3,f_{b}=0.4$. The blue color represents the A blocks, the red color represents the B blocks.

Figure 2 shows the morphologies of the ABA star polymer blend without nanoparticles in bulk. In Figure 2a, it can be seen that the phase separation is not obvious when $f_{a1}=f_{a2}=0.13,f_{b}=0.74$. While as is shown in Figure 2b, the ABA star polymer formed bicontinuous structures when $f_{a1}=f_{a2}=0.2,f_{b}=0.6$. Furthermore, when $f_{a1}=f_{a2}=0.3,f_{b}=0.4$, the ABA polymer formed cylindrical structures as hexagonally ordered, which were randomly arranged, as shown in Figure 2c.

**Figure 3:**
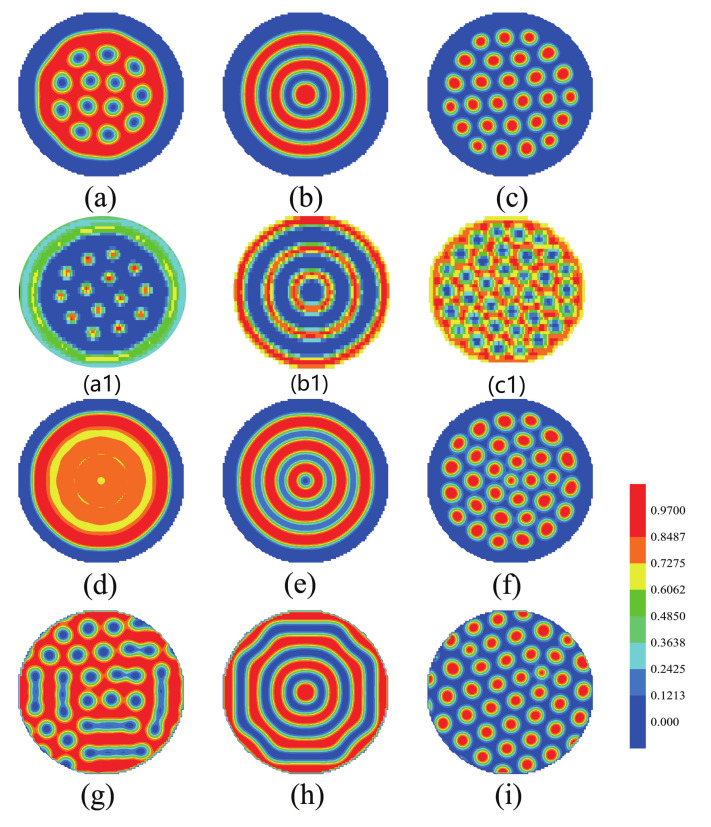
(**a**–**c**) The morphologies of the mixture of ABA star polymer and nanoparticles confined in a sphere with soft inner surface; the blue color represents the A blocks and the brushes, the red color represents the B blocks. The nanoparticles disperse in A domain. (**a1**–**c1**) The morphologies of nanoparticles; the red color represents the nanoparticles. (**d**–**f**) The morphologies of ABA star polymer without nanoparticles confined in a sphere with soft inner surface; the blue color represents the A blocks and the brushes, the red color represents the B blocks. (**g**–**i**) The morphologies of the mixture of ABA star polymers and nanoparticles confined in a hard sphere. the blue color represents the A blocks and the brushes, the red color represents the B blocks. The left column represents $f_{a1}=f_{a2}=0.13,f_{b}=0.74$; the middle column represents $f_{a1}=f_{a2}=0.2,f_{b}=0.6$; the right column represents $f_{a1}=f_{a2}=0.3,f_{b}=0.4$.

Figure 3a–c show the morphologies of the mixture of the ABA star polymer and nanoparticles confined in a sphere with soft inner surface when the fraction of the B blocks is changed. When $f_{a1}=f_{a2}=0.13,f_{b}=0.74$, the A blocks formed the cylindrical structures which dispersed in the B domains. When the blocks had equal lengths, both the A and B blocks formed the concentric lamellae structures. And when $f_{a1}=f_{a2}=0.3,f_{b}=0.4$, the B blocks formed the cylindrical structures which dispersed in the A domains. Figure 3a1–c1 show the morphologies of nanoparticles, the red color represents the nanoparticles. The nanoparticles appeared cylindrical in Figure 3a1, concentric lamellae in Figure 3b1 and dispersed phase in Figure 3c1 with decreasing the volume fraction of B blocks. Figure 3d–f show the morphologies of the ABA star polymer confined in a soft sphere with changing the fraction of blocks. When the fraction of the A blocks was relatively small, the phase separation of the ABA star polymer was not obvious, as shown in Figure 3d. When the blocks were asymmetric, the ABA star polymer formed the concentric lamellae in Figure 3e. With continuously decreasing the fraction of the B blocks, the B bloks formed the cylinders in Figure 3f. Figure 3g–i show the morphologies of the ABA star polymer and nanoparticles confined in a hard sphere with changing the fraction of the B blocks. When $f_{a1}=f_{a2}=0.13,f_{b}=0.74$, the mixture phase separated the disordered structure. When $f_{a1}=f_{a2}=0.2,f_{b}=0.6$, the length of the A block is close to B block, the mixture formed concentric lamellae with a bit of a defect. when $f_{a1}=f_{a2}=0.3,f_{b}=0.4$, the B blocks formed cylinders phase.

The morphologies in Figure 3a–c containing the nanoparticles were compared with those in Figure 3d–f without the nanoparticles. As Figure 3a,d, when the fraction of the A blocks was relatively small in Figure 3a, the system formed ordered cylinders after adding the nanoparticles instead of the disordered phase in Figure 3d. With increasing the fraction of the A blocks to $f_{a1}=f_{a2}=0.2,f_{b}=0.6$, the lengths of the A and B blocks is almost equal. The phase separation of the system was complete and formed the concentric lamellae both in Figure 3b,e. With continuously increasing the fraction of the A blocks, the B blocks formed cylinders with the different domain sizes in Figure 3c,f, it is due to the gather of adding nanoparticles. The competition effect of the nanoparticles entropy and the wetting energy with A blocks could improve the different domain sizes.

The morphologies in Figure 3a–c with the mixture of ABA star polymer and nanoparticles being confined in a soft sphere were compared with those in Figure 3g–i with the mixture being confined in a hard sphere. It is observed that the ABA star polymer and nanoparticles could form regular and ordered structures in advance when the mixture was confined in a soft sphere in Figure 3a–c. When the fraction of the A blocks was relatively small in Figure 3g, the star polymer formed disordered structure with the rigid confinement, which could increase the interface energies. When the fraction of the A blocks was relatively big in Figure 3h, the B blocks formed concentric lamellae with a bit of a defect in confinement. In comparison, the mixture formed ordered structure of concentric ring when confined in a soft sphere in Figure 3b due to the wetting effect of the brushes. Figure 3i showed the cylindrical structures as hexagonally ordered just like it in the bulk.

**Figure 4:**
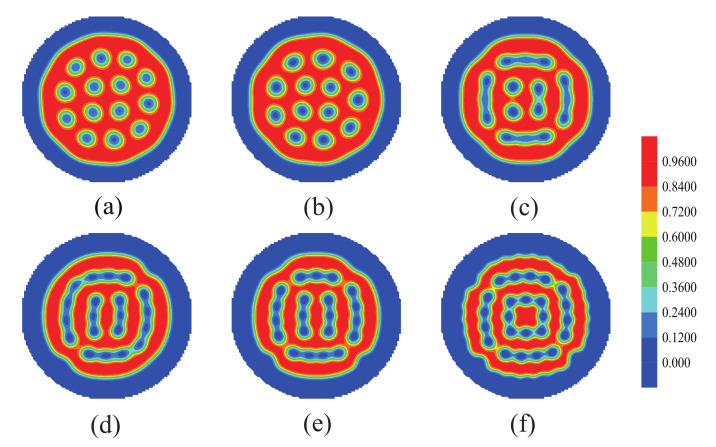
The morphologies of ABA star polymer and nanoparticles with increasing the volume fraction of nanoparticle $f_{p}$ for nanoparticle radius $R_{p}/R_{g}=0.75$ and $f_{a1}=f_{a2}=0.13,f_{b}=0.74$. (**a**) $f_{p}=0.13$, (**b**) $f_{p}=0.15$, (**c**) $f_{p}=0.19$, (**d**) $f_{p}=0.24$, (**e**) $f_{p}=0.25$, (**f**) $f_{p}=0.28$. The blue color represents the A blocks and the brushes, the red color represents the B blocks.

Figure 4 shows the morphologies of ABA star polymer and nanoparticles with increasing the volume fraction of nanoparticle $f_{p}$ for nanoparticle radius $R_{p}/R_{g}=0.75$ and $f_{a1}=f_{a2}=0.13,f_{b}=0.74$. In Figure 4a, when $f_{p}=0.13$, the mixture formed cylinders, $C_{4-10}$, where 4 represents the number of cylinders in the inner layer and 10 represents the number of cylinders in the outer layer. In Figure 4b, when $f_{p}=0.15$, the mixture also formed ordered cylinders, but that is $C_{4-9}$. As continuously increasing the volume fraction of nanoparticles in Figure 4c–f, the mixture formed the multiple-continuous phases. When these multiple-continuous phases were compared, the structures in Figure 4c had rotational symmetry, the morphologies of the mixture in Figure 4d had center symmetry and the structures in Figure 4e,f had both rotational symmetry and center symmetry. This is because the increase in nanoparticle volume fraction resulted in the increase in the steric repulsion and the interactional energies between the nanoparticles and the blocks. In order to decrease the energies, the multiple-continuous phases were formed, which led to the conformational entropy loss because the ABA star polymer was in a confined environment. In addition, it was observed that the surface of homopolymer brushes was deformed to decrease the energies and match the morphologies of ABA star polymer and nanoparticles.

**Figure 5:**
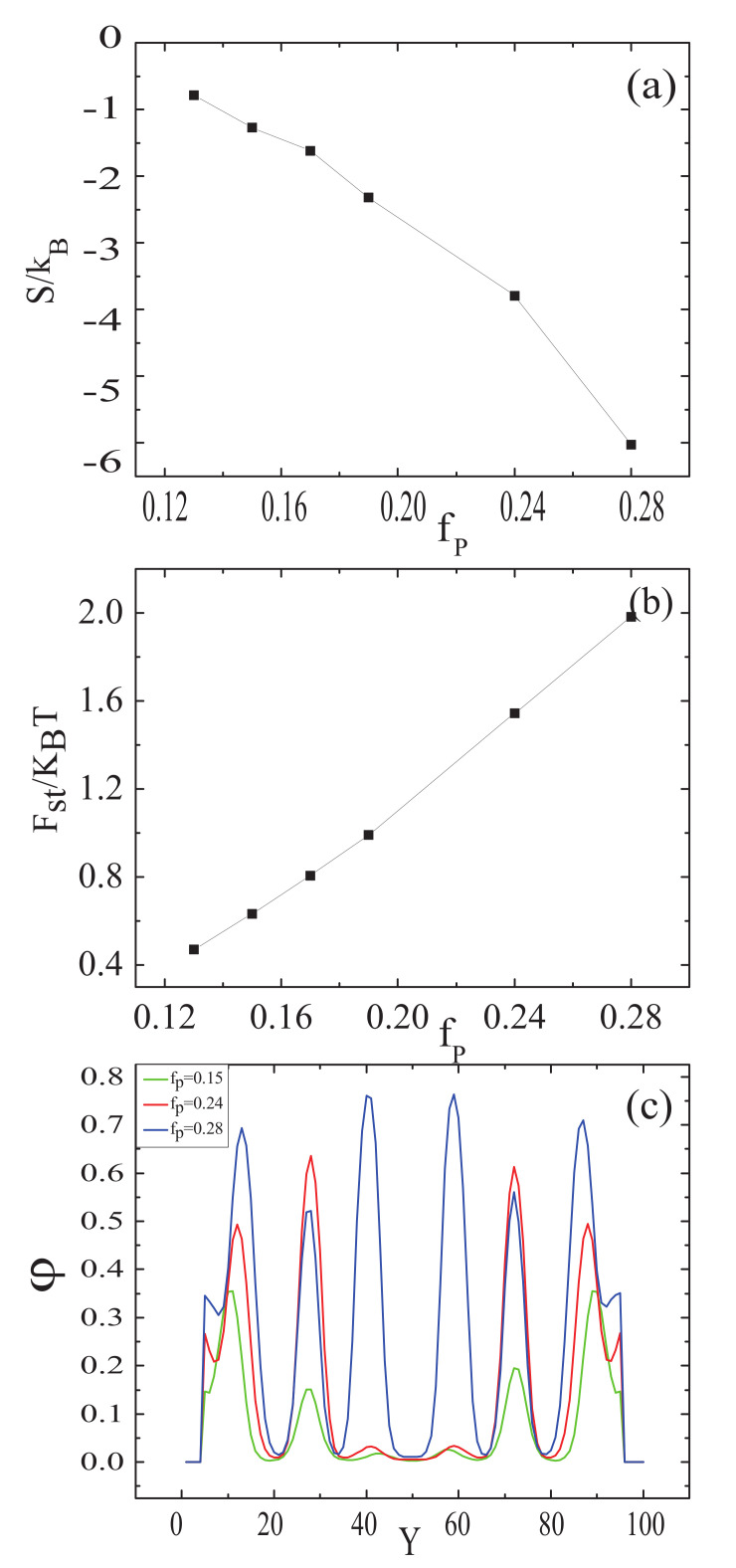
(**a**) The entropy of the nanoparticles, $S/k_{B}$, as a function of the volume fraction of nanoparticles, $f_{p}$, when $f_{a1}=f_{a2}=0.13,f_{b}=0.74$; (**b**) The steric packing interaction of the nanoparticles, $F_{st}/K_{B}T$, as a function of the volume fraction of nanoparticles, $f_{p}$, when $f_{a1}=f_{a2}=0.13,f_{b}=0.74$; (**c**) Nanoparticles density distributions along the perpendicular line through the center of the sphere with $f_{a1}=f_{a2}=0.13,f_{b}=0.74$, the green, red and blue colors represent $f_{p}=0.15$, $f_{p}=0.24$ and $f_{p}=0.28$, respectively.

Allowing the nanoparticles to interact with the B blocks, the morphologies of the nanoparticles were similar to those of the A blocks. To obtain the comprehensive information on the morphologies of the mixture, the entropy and the steric packing interaction of nanoparticle as a function of the nanoparticle volume fraction, and the nanoparticle density distributions along the perpendicular line through the center of the sphere with $f_{a1}=f_{a2}=0.13,f_{b}=0.74$ are plotted in Figure 5. It can be seen from Figure 5a that the entropy of nanoparticle decreases with the increase in nanoparticle volume fraction. The entropy decreases remarkably from $f_{p}=0.24$ to $f_{p}=0.28$ due to the formation of multiple-continuous phases. Figure 5b shows that the steric packing interaction of nanoparticle increases as increasing the nanoparticle volume fraction. The curve is nearly similar to a straight line, indicating that the change of the steric packing interaction was proportional to the change of nanoparticle volume fraction. It can also be seen from Figure 5b that the steric packing energy slightly increases after $f_{p}=0.19$, which could make the nanoparticles separate from each other, and thus the multiple-continuous structures were formed. In Figure 5c, the first characteristic is the intensities of some pecks strengthen with increasing the nanoparticle volume fraction. However, the intensities of $f_{p}=0.24$ are stronger than those of $f_{p}=0.28$ when 25≤Y≤33 and 68≤Y≤77. This is because the nanoparticle concentration of $f_{p}=0.24$ are much higher than that of $f_{p}=0.28$, as in Figure 4d,f. The second characteristic is the widths of the outermost lamellae and the nanoparticle concentration increases with the increase in nanoparticle volume fraction, which can be seen from the two sides of Figure 5c. The most outstanding characteristic is that the intensities of $f_{p}=0.28$ are much stronger than those of others at Y=30 and Y=60, in that the nanoparticle concentration are much higher near at the center of sphere, which could be seen in Figure 4f.

**Figure 6:**
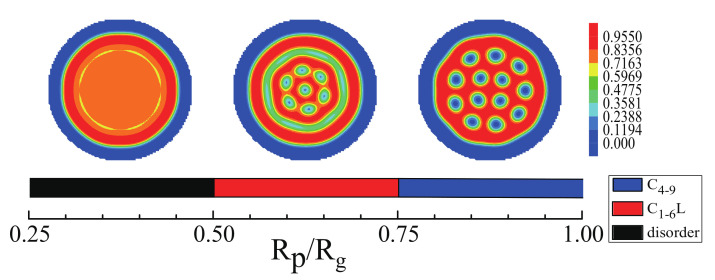
The regions of different morphologies of ABA star polymer and nanoparticles mixtures as a function of the nanoparticle radius for $f_{p}=0.15$ and $f_{a1}=f_{a2}=0.13,f_{b}=0.74$. The blue color represents the A blocks and the brushes, the red color represents the B blocks.

Next, the morphologies of ABA star polymer and nanoparticles with the increase of the nanoparticle radius were considered. Figure 6 shows the regions of different structures formed by ABA star polymer and nanoparticles when changing the nanoparticle radius for $f_{p}=0.15$ and $f_{a1}=f_{a2}=0.13,f_{b}=0.74$. When $0.25\leq R_{p}/R_{g}<0.5$, the phase separation of the mixture was not obvious. While when $0.5\leq R_{p}/R_{g}<0.75$, the mixture of cylinder and lamellae, $CL_{1-6}$ was formed, and when $0.75\leq R_{p}/R_{g}<1.0$, the ordered cylinders, $C_{4-9}$ were obtained. However, with increasing the nanoparticle radius, the mixture formed disordered morphologies. The top layer of the figure represented the structures of the corresponding region. Form these results, it could be concluded that the nanoparticle radius played an important role in controlling the morphologies of the mixture and the increase in the nanoparticle radius could urge the nanoparticle into the cylinders. Similar conclusion was presented by Zhang et al. [52].

**Figure 7:**
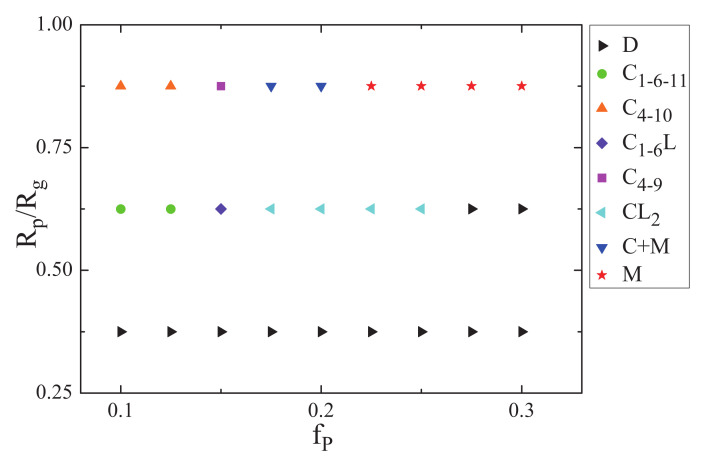
The phase diagram of the mixture of the ABA star polymer and nanoparticles as a function of the nanoparticle volume fraction $f_{p}$ and the nanoparticle radius $R_{p}/R_{g}$ with $f_{a1}=f_{a2}=0.13,f_{b}=0.74$, the righttriangle, circle, uptriangle, diamond, square, lefttriangle, downtriangle and star represent the phase separation of the mixture, cylinder $C_{1-6-11}$, cylinder $C_{4-10}$, mixture of cylinders and lamellae $C_{1-6}L$, cylinder $C_{4-9}$, mixture of cylinders and lamellae $CL_{2}$, mixture of cylinders and multiple-continuous phases C+M and multiple-continuous phases *M*, respectively.

Allowing the nanoparticles to interact with the B blocks, the ABA star polymer and nanoparticles were confined in a sphere with soft inner surface. In order to systemically investigate the effect of the nanoparticle volume fraction and the nanoparticle radius on the phase behaviors of the ABA star polymer and nanoparticles, the phase diagram of the mixture of the ABA star polymer and nanoparticles were plotted as a function of the nanoparticle volume fraction $f_{p}$ and the nanoparticle radius $R_{p}/R_{g}$ with $f_{a1}=f_{a2}=0.13,f_{b}=0.74$, showed in Figure 7. Obviously, a variety of structures occurred which were composed of the ABA star polymer and nanoparticles. When the nanoparticles radius was small, the phase separation of the mixture was not obvious, whereas, when both the radius and the volume fraction of nanoparticles were large, the multiple-continuous phases were formed. However, when the nanoparticles volume fraction was small, the mixture mainly formed cylindrical structures.

### 3.2. Nanoparticles Interact with the A Blocks and the Homopolymer Brushes

**Figure 8:**
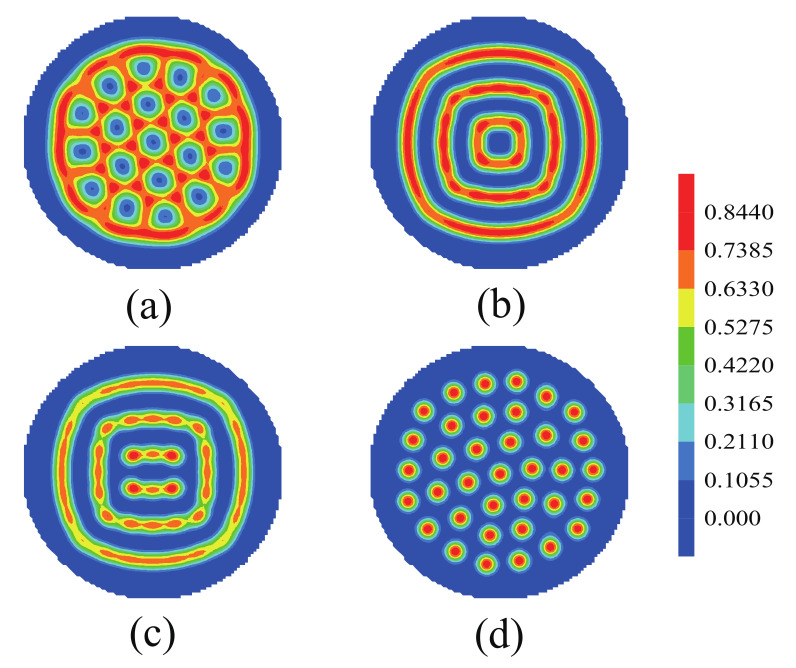
The morphologies of the nanoparticles as a function of the volume fraction of each block when we allow nanoparticles to interact with both A blocks and homopolymer brushes. (**a**) $f_{a1}=f_{a2}=0.18,f_{b}=0.64$, (**b**) $f_{a1}=f_{a2}=0.3,f_{b}=0.4$, (**c**) $f_{a1}=f_{a2}=0.33,f_{b}=0.34$, (**d**) $f_{a1}=f_{a2}=0.41,f_{b}=0.18$. The blue color represents the A blocks and the brushes, the red color represents the nanoparticles.

In the second part, the nanoparticles were allowed to interact with both the A blocks and the homopolymer brushes with $\chi_{ap}N=\chi_{brp}N=20.0,\chi_{bp}N=0$, which the nanoparticles wet to the B blocks. When the nanoparticles interact with both the A blocks and the homopolymer brush, the particles can coexist with the B blocks. That means that the particles are distributed in the domain structure formed by the B blocks. For clear explanation, the morphologies of the nanoparticles are shown in Figure 8. The red color represents the nanoparticles.

Firstly, the morphologies of the nanoparticles with changing the fraction of each block were discussed. It is worth mentioning that the morphologies of the B blocks are similar to those of the nanoparticles, the red color represents the morphologies of both the B blocks and the nanoparticles, and the blue color represents the morphologies of the A blocks. In Figure 8a, the A blocks form cylinders dispered in the domain of the nanoparticles and the B blocks. Figure 8b shows the concentric lamellae formed by the nanoparticles and the B blocks. Figure 8c shows two types of lamellae, with one type being two concentric lamellae and the other being two parallel lamellae. Figure 8d shows the cylinders formed by the nanoparticles and the B blocks, which disperse in the A domains. From Figure 8a–d, the morphologies of the nanoparticles from disordered to ordered with increasing the fraction of the A blocks, which appeared a concentric lamellae phase, a square-like shape concentric lamellae phase and a cylinders phase. That phenomenon due to the repulsive force of the nanoparticles and the A blocks increased with increasing the fraction of A blocks. The interface energy between the nanoparticles and the brushes also increased, it makes the nanoparticles gather to the center of the sphere.

**Figure 9:**
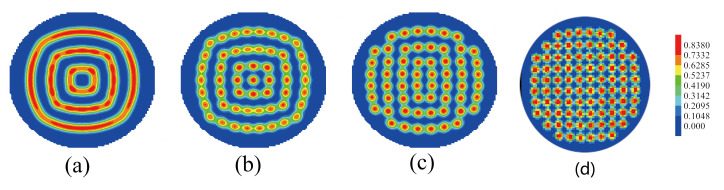
The morphologies of the nanoparticles with increasing the nanoparticle volume fraction $f_{p}$ for nanoparticle radius $R_{p}/R_{g}=0.75$ and $f_{a1}=f_{a2}=0.3,f_{b}=0.4$. (**a**) $f_{p}=0.15$, (**b**) $f_{p}=0.20$, (**c**) $f_{p}=0.23$, (**d**) $f_{p}=0.28$. The blue color represents the A blocks and the brushes, the red color represents the nanoparticles.

The effect of the nanoparticle volume fraction on the morphologies of ABA star polymer and nanoparticles was investigated, allowing the nanoparticles to interact with both the A blocks and the homopolymer brushes. Figure 9a–c show the morphologies of the nanoparticles with the increase of the nanoparticle volume fraction for nanoparticle radius $R_{p}/R_{g}=0.75$. With increasing the nanoparticle volume fraction, the morphologies of the nanoparticles formed the concentric lamellae, the concentric-square lamellae and column dispersion, which are plotted in Figure 9. In Figure 9a, the concentric-square lamellae formed when $f_{p}=0.15$. This is because the wetting effect of the homopolymer brushes and the geometrical shape of confinement could together impel the nanoparticles to form the concentric lamellae. When $f_{p}=0.20$, the squarely multiple-continuous phase was obtained, which had both rotational symmetry and center symmetry. It is attributed to that the quick increase in the steric packing interaction of a single nanoparticle could push the nanoparticles apart. While when the nanoparticle volume fraction increased to 0.23, the continuous rectangle phase was formed. And the surfaces of the A blocks and the homopolymer brushes were deformed in order to decrease the energies and match the morphologies of the nanoparticles. When $f_{p}=0.28$, the nanoparticles occurred column dispersion phase. When the nanoparticles interact with both the A block and the homopolymer brush, the particles can coexist with the B block. That means that the particles are distributed in the domain structure formed by the B block. When the volume fraction of the nanoparticles increased, the arrangements the nanoparticles formed a variety of “square” structure. Because of the steric packing interaction of naonoparticles increased, the nanoparticles go to disperse. While the interface energy between the nanoparticles and the A block increased, it makes the nanoparticles to gather. The interface energy between the nanoparticles and the brushes also increased, it makes the nanoparticles gather to the center of the sphere. This “disperse-gather” incessant competitions lead to the nanoparticles formed striped arrangement structure.

**Figure 10:**
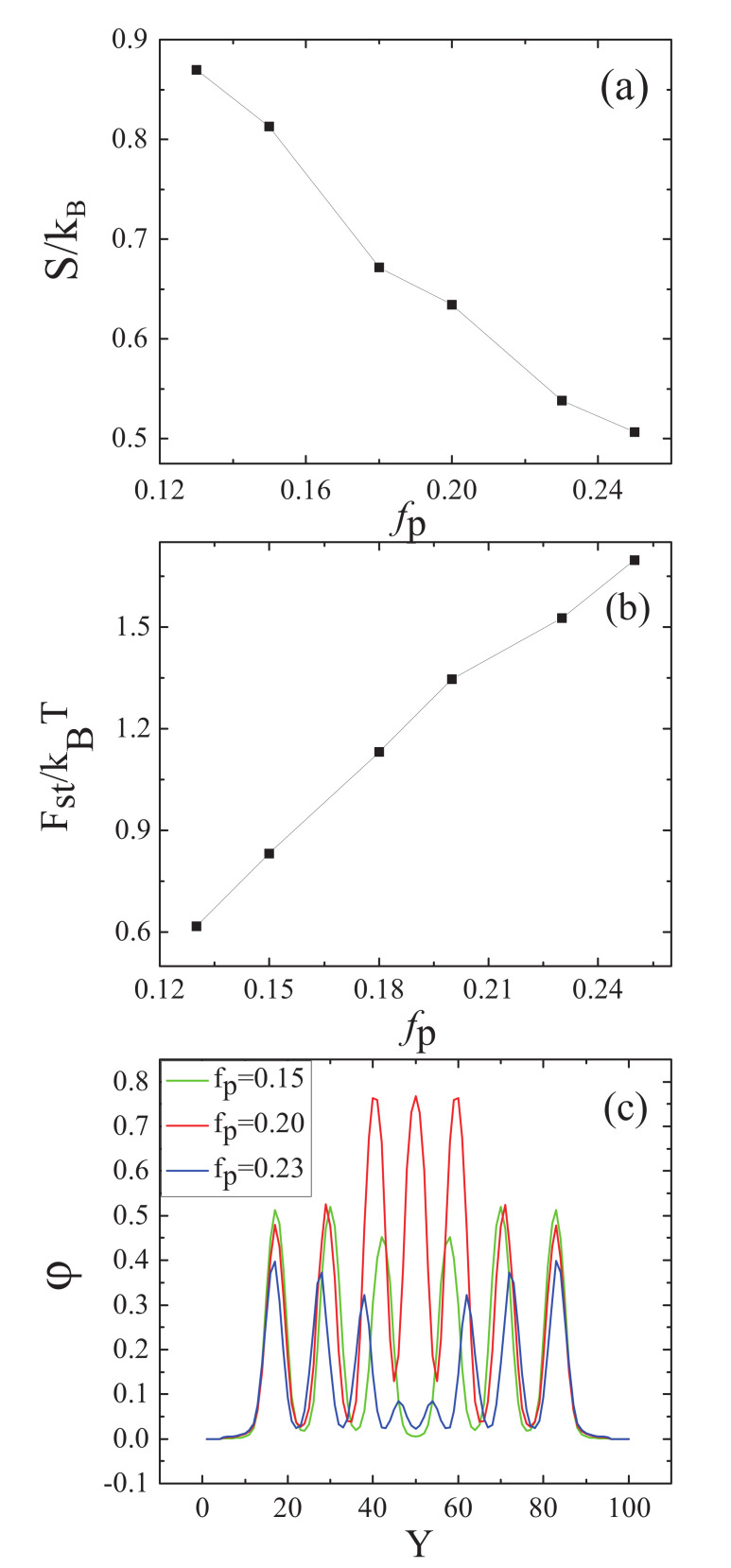
(**a**) The entropy of the nanoparticles, $S/k_{B}$, as a function of the nanoparticles volume fraction $f_{p}$, when $f_{a1}=f_{a2}=0.13,f_{b}=0.74$; (**b**) The steric packing interaction of the nanoparticles, $F_{st}/K_{B}T$, as a function of the nanoparticles volume fraction $f_{p}$, when $f_{a1}=f_{a2}=0.3,f_{b}=0.4$; (**c**) Nanoparticles density distributions along the perpendicular line through the center of the sphere with $f_{a1}=f_{a2}=0.3,f_{b}=0.4$, the green, red and blue colors represent $f_{p}=0.15$, $f_{p}=0.20$ and $f_{p}=0.23$, respectively.

The entropy and the steric packing interaction of nanoparticles were plotted as a function of the nanoparticles volume fraction, and nanoparticle density distributions along the perpendicular line through the center of the sphere with $f_{a1}=f_{a2}=0.3,f_{b}=0.4$, as shown in in Figure 10. From Figure 10a, it can be seen that the entropy of nanoparticles decreased with increasing nanoparticles volume fraction and the entropy remarkably decreased as $f_{p}=0.15$ to $f_{p}=0.18$. The mixture formed the multiple-continuous structures, which could lead to the entropy loss in the confined environment. Figure 10b shows that the steric packing interaction of nanoparticles increased with increasing the nanoparticles volume fraction. The curve was nearly similar to a straight line before $f_{p}=0.20$, which indicated that the change of steric packing interaction was proportional to the change of the nanoparticles volume fraction. After $f_{p}=0.20$, the increasing rate of the steric packing interaction of nanoparticles with the increase of the nanoparticles volume fraction slowed down due to the effect of the nanoparticles separation. Figure 10c shows the nanoparticles density distributions along the perpendicular line through the center of the sphere. The first characteristic is that the intensities of some pecks weakened with increasing the nanoparticles volume fraction, due to the difference in the situation of the interaction between the nanoparticles and the B blocks. The second characteristic is that the intensities of $f_{p}=0.20$ are much stronger than those of the other two situations in the center of the sphere, because of the higher nanoparticles concentration in this region when $f_{p}=0.20$, as shown in Figure 9a–c. The third characteristic is that the widths of the outermost layer lamellae and the nanoparticles concentration decrease with the increase of nanoparticles volume fraction, which can be seen from the two sides of Figure 10c.

**Figure 11:**
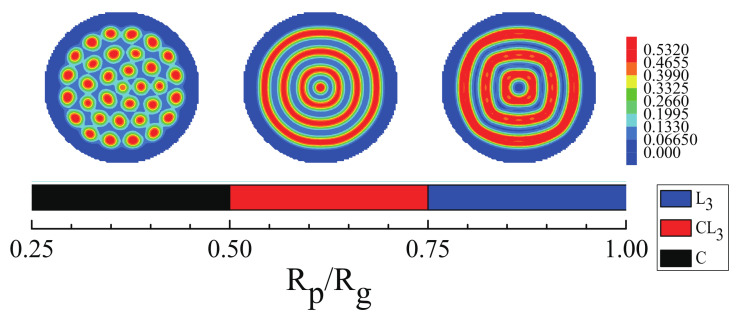
The regions of different morphologies of ABA star polymers and nanoparticles as a function of the nanoparticles radius with $f_{p}=0.15$ and $f_{a1}=f_{a2}=0.3,f_{b}=0.4$. The blue color represents the A blocks and the brushes, the red color represents the B blocks.

Then, the effect of the nanoparticle radius on the mixture of morphologies of ABA star polymers and nanoparticles were studied. Figure 11 shows the regions of different structures formed by the mixture with changing the nanoparticle radius for $f_{p}=0.15$ and $f_{a1}=f_{a2}=0.3,f_{b}=0.4$. When $0.25\leq R_{p}/R_{g}<0.5$,, the B blocks formed cylinders which were non-hexangularly arranged in the sphere and the domain sizes of cylinders were not uniform. While, when $0.5\leq R_{p}/R_{g}<0.75$, the mixture of cylinder and lamellae, $CL_{3}$, was formed, and when $0.75\leq R_{p}/R_{g}<1.0$, the concentric lamellae, $L_{3}$, was obtained. However, with increasing the nanoparticle radius, the mixture of ABA star triblock copolymers and nanoparticles formed disordered morphologies. The top layer of the figure represented the structures of the corresponding region.

**Figure 12:**
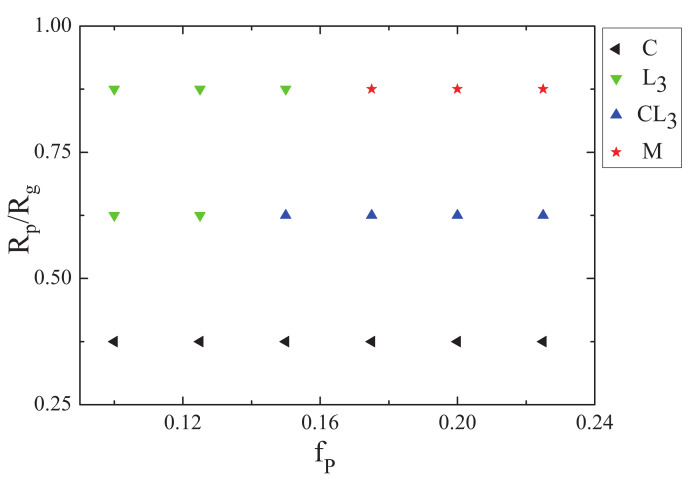
The phase diagram of the mixture of ABA star polymers and nanoparticles as a function of the nanoparticles volume fraction $f_{p}$ and the nanoparticle radius $R_{p}/R_{g}$ with $f_{a1}=f_{a2}=0.3,f_{b}=0.4$, the lefttriangle, downtriangle, uptriangle, and star represent the cylindrical structurea, concentric lamellaes $L_{3}$, mixture of cylinders and lamellae $CL_{3}$ and multiple-continuous phases *M*, respectively.

In addition, allowing the nanoparticle to interact with both the A blocks and the homopolymer brushes, the effect of the nanoparticle volume fraction and nanoparticle radius on phase behaviors of ABA star polymers confined in a sphere with soft inner surface were investigated. The phase diagram of the mixture of the ABA star polymers and nanoparticles as a function of the nanoparticles volume fraction $f_{p}$ and the nanoparticle radius $R_{p}/R_{g}$ with $f_{a1}=f_{a2}=0.3,f_{b}=0.4$ was plotted, shown in Figure 12. When the nanoparticle radius was small, the mixture of the ABA star polymers and nanoparticles mostly formed cylindrical structures, and when the nanoparticles volume fraction was small, the mixture mostly formed concentric lamellae. However, when both the nanoparticle radius and nanoparticle concentration were large, the multiple-continuous phases were formed, which was similar to the situation of the interaction between the nanoparticles and the B blocks.

### 3.3. The Effect of Sphere Radius on the Morphologies of the Mixture of ABA Star Polymers and Nanoparticles

**Figure 13:**
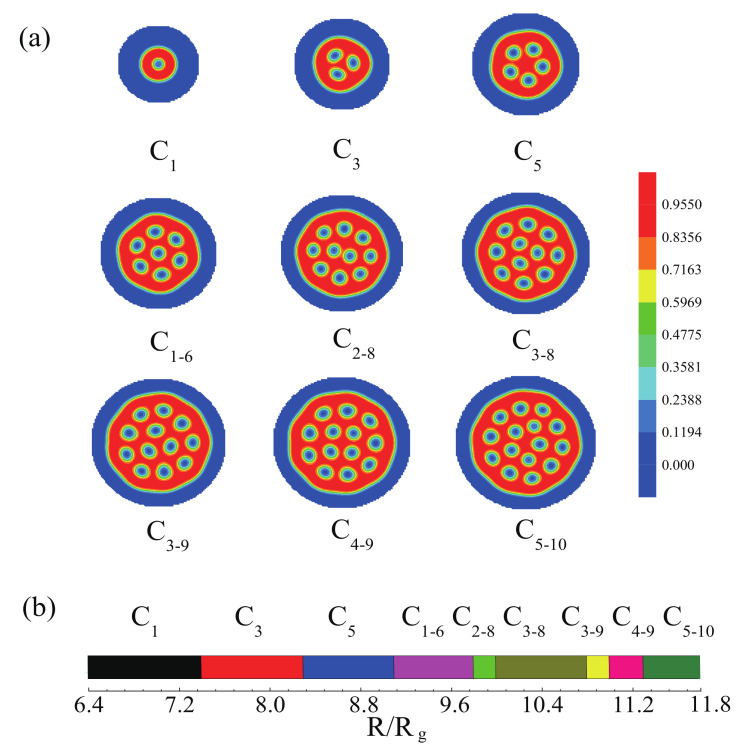
(**a**) The morphologies observed in the mixture of ABA star polymers and nanoparticles with $f_{a1}=f_{a2}=0.13,f_{b}=0.74$, $f_{p}=0.15$ and $R_{p}/R_{g}=0.75$ and the interaction between the nanoparticles and the B blocks, under the spherical confinement with varying the radius of sphere; (**b**) The phase sequence of morphologies as a function of $R/R_{g}$. The blue color represents the A blocks and the brushes, the red color represents the B blocks.

In this part, the effect of sphere radius on the morphologies of the mixture of ABA star polymers and nanoparticles was considered, which was a main factor to control the phase behaviors of the mixture in this system. Figure 13a shows the morphologies of the mixture with increasing the sphere radius for $f_{a1}=f_{a2}=0.13,f_{b}=0.74$ and the interaction between the nanoparticles and the B blocks. It can be seen that the mixture formed different cylinders. In Figure 13a, the number of cylinders increased with the increase of the sphere radius. This is because the domain size was determined by the confinement environment. In addition, the mixture formed lamellae in the sphere surface in order to decrease the interface energies. The sphere boundary made the 2D phase sequence, $C_{1}\to C_{3}\to C_{5}\to C_{1-6}\to C_{2-8}\to C_{3-8}\to C_{3-9}\to C_{4-9}\to C_{5-10}$. Figure 13b represents the phase sequence of the morphologies as a function of $R/R_{g}$, which was different from that in the hard spherical confinement and the lateral hexagonal confinement, such as $C_{2-8}$, which was not obtained in the hard spherical confinement, and the $C_{2-8}$ was metastable morphology in the lateral hexagonal confinement.

## 4. Conclusions

In this paper, the phase behaviors of ABA star polymer and nanoparticles confined in a sphere with soft inner surface using the SCFT and DFT were investigated. Two kinds of nanoparticles were considered. One kind of the nanoparticles could interact with the B blocks and the other could interact with both the A blocks and the homopolymer brushes. With changing the block ratio, the concentration and the radius of nanoparticles to study the phase behaviors of the ABA star polymer and nanoparticles mixtures confined in the soft sphere were systematically studied. By means of simulation, a variety of interesting structures and many conclusions were obtained. Firstly, the copolymers could form ordered structures in advance with nanoparticles when comparing to those without nanoparticles. Secondly, whether the nanoparticles interacted with the B blocks or with both the A blocks and the homopolymer brushes, the multiple-continuous structures were formed when increasing the nanoparticle volume fraction. And the entropy and the steric repulsive interaction of nanoparticles exhibited the same trend to disperse with increasing the nanoparticle volume fraction, which indicated that the effect of the nanoparticles concentration played a more important role than the interaction between the nanoparticles and the polymers. As for the nanoparticles interacted with both the A blocks and the homopolymer brushes separated from each othermuch earlier than those interacting with B blocks. Thirdly, the phase diagram of copolymer-nanoparticle mixtures as a function of the volume fraction and the radius of nanoparticles was plotted. It was found that the two factors had competition effect on the morphologies of the mixture. Finally, the soft confinement effect on the phase behaviors of the system was studied and the phase sequence of the mixture confined in a sphere with soft inner surface was obtained, These investigations showed that interesting morphologies and structures could be obtained by tuning the main parameters, such as the volume fraction, the radius of nanoparticles, and the interaction between the nanoparticles and the blocks. And these variable parameters had the competition effect of “dispersed-gather” incessant on the morphologies of the mixture. The results in this work are expected to provide an effective way to fabricate the newly multifunctional materials.
